# PERCEPTIONS OF INDIVIDUALS WHO CONFRONT AGEISM

**DOI:** 10.1093/geroni/igz038.309

**Published:** 2019-11-08

**Authors:** Michelle Horhota, Alison L Chasteen, Jessica Crumley-Branyon

**Affiliations:** 1 Furman University, Greenville, South Carolina, United States; 2 University of Toronto, Toronto, Canada

## Abstract

What are the consequences for older adults who confront ageism? We compared young (n=316), middle-aged (n=464), and older adults’ (n=273) perceptions of an older target who confronts the perpetrator of an ageist action. Participants read a vignette about a pedestrian offering unwanted help to an older woman crossing the street. We manipulated the type of ageism (benevolent or hostile), the reaction of the older target (acceptance, moderate confrontation or strong confrontation) and assessed how impressions of warmth, competence and overall impression of the target changed over time. Type of Ageism x Reaction x Time interactions emerged for all three variables. In the hostile condition, a strong confrontation resulted in the target being rated as less warm, more competent, and the overall impression decreased over time. In contrast, a moderate confrontation increased perceptions of warmth, competence and overall ratings of the target. In the benevolent condition, a strong confrontation decreased perceptions of the target’s warmth, competence and overall impression. Moderate confrontation increased perceptions of target competence but did not change perceptions of warmth or overall impression. Targets that accepted the ageist act were rated lower on warmth for both hostile and benevolent conditions. Competence ratings were not affected. However, targets that accepted benevolent ageism experienced a cost to their overall impression. Taken together, these results suggest that when confronting ageism, older adults should take a moderate approach. When participants perceived the target’s reaction to be incommensurate with the offer of help, the target was viewed more negatively overall.

